# O_2_ Level Controls Hematopoietic Circulating Progenitor Cells Differentiation into Endothelial or Smooth Muscle Cells

**DOI:** 10.1371/journal.pone.0005514

**Published:** 2009-05-13

**Authors:** Nicolas Berthelemy, Halima Kerdjoudj, Pierre Schaaf, Christine Prin-Mathieu, Patrick Lacolley, Jean-François Stoltz, Jean-Claude Voegel, Patrick Menu

**Affiliations:** 1 Group of Bioengineering (UMR CNRS 7561), UHP-Nancy 1, Faculté de Médecine, Vandoeuvre-lès-Nancy, France; 2 Institut Charles Sadron (UPR 22, CNRS), Strasbourg, France; 3 CHU Nancy and UHP-Nancy 1, Faculté de Médecine, Vandoeuvre-lès-Nancy, France; 4 Institut National de la Santé et de la Recherche Médicale, (INSERM Unité 961), UHP-Nancy 1, Faculté de Médecine, Vandoeuvre-lès-Nancy, France; 5 Institut National de la Santé et de la Recherche Médicale, (INSERM Unité 977), Université de Strasbourg, Faculté de chirurgie dentaire, Strasbourg, France; Tufts University, United States of America

## Abstract

**Background:**

Recent studies showed that progenitor cells could differentiate into mature vascular cells. The main physiological factors implicated in cell differentiation are specific growth factors. We hypothesized that simply by varying the oxygen content, progenitor cells can be differentiated either in mature endothelial cells (ECs) or contractile smooth muscle cells (SMCs) while keeping exactly the same culture medium.

**Methodology/Principal Findings:**

Mononuclear cells were isolated by density gradient were cultivated under hypoxic (5% O_2_) or normoxic (21% O_2_) environment. Differentiated cells characterization was performed by confocal microscopy examination and flow cytometry analyses. The phenotype stability over a longer time period was also performed. The morphological examination of the confluent obtained cells after several weeks (between 2 and 4 weeks) showed two distinct morphologies: cobblestone shape in normoxia and a spindle like shape in hypoxia. The cell characterization showed that cobblestone cells were positive to ECs markers while spindle like shape cells were positive to contractile SMCs markers. Moreover, after several further amplification (until 3^rd^ passage) in hypoxic or normoxic conditions of the previously differentiated SMC, immunofluorescence studies showed that more than 80% cells continued to express SMCs markers whatever the cell environmental culture conditions with a higher contractile markers expression compared to control (aorta SMCs) signature of phenotype stability.

**Conclusion/Significance:**

We demonstrate in this paper that in vitro culture of peripheral blood mononuclear cells with specific angiogenic growth factors under hypoxic conditions leads to SMCs differentiation into a contractile phenotype, signature of their physiological state. Moreover after amplification, the differentiated SMC did not reverse and keep their contractile phenotype after the 3^rd^ passage performed under hypoxic and normoxic conditions. These aspects are of the highest importance for tissue engineering strategies. These results highlight also the determinant role of the tissue environment in the differentiation process of vascular progenitor cells.

## Introduction

During embryogenesis, vasculogenesis is one of the first initiated processes. Conversely in the adult, the new vessels formation is initiated from the existent blood vessel ramifications. Data accumulated in recent years indicate that the circulating mononuclear cell (MNCs) fractions contain a population of bone marrow derived cells called progenitor cells that contribute to the neovascularization of injured vessels. Different authors [1]–[5] suggested that these progenitor cells could differentiate in the presence of different specific cytokines and angiogenic growth factors (vascular endothelial growth factor (VEGF), platelet derived growth factor BB (PDGF-BB)…), into mature and functional endothelial (ECs) or vascular smooth muscle (SMCs) cells depending on the added specific growth factors. During wound healing, ischemia, vascular wall remodelling or tumour development, the formation of new blood vessels is preceded by the recruitment of MNCs at the injured sites which further promote vasculogenesis [6]–[9]. Various authors investigated also the role of the oxygen concentration on stem cells differentiation and it was shown that hypoxia increased the production of angiogenic growth factors such as transforming growth factor β1, PDGF-BB and VEGF [10]–[12]. The main physiological factors implicated in cell differentiation are angiogenic growth factors (*i.e*: VEGF, bFGF and IGF) [2], [3], [13] and a decrease of the oxygen level in the tissue (hypoxia) [5]. Oxygen plays a main role in physiological and pathological states [14]; it is a potent biochemical signalling molecule with important regulation properties for cellular behaviour (migration, differentiation, proliferation…) [15]–[17]. However, the possible involvement of hypoxia in MNCs differentiation into SMCs has never been demonstrated and even mentioned up to now.

We hypothesized here that the only oxygen concentration tuning combined with growth factors favouring ECs differentiation (VEGF, FGF, EGF, IGF) [18] allow the differentiation of circulating progenitor cells into mature ECs or contractile SMCs, characteristic of mature vascular cells found *in vivo*.

We demonstrate that progenitor cells isolated from rabbit fraction cultivated onto specifically coated solid substrates (either by type I collagen: a compound of the arterial wall and known as an ideal substrate for adhesion and proliferation of vascular smooth muscle cells in vitro [2] or by a Polyelectrolyte Multilayered Film architecture which previously demonstrated an important speeding up of endothelial progenitor cells differentiation into mature and functional endothelial cells [19]) in normoxic conditions (21% O_2_ atmosphere or 151 mmHg) lead to mature ECs and to SMCs when cultivated in exactly the same medium but under moderate hypoxic conditions (5% O_2_ or 36 mmHg). Whereas it is well established that the culture of mature SMCs leads to a decrease of contractile markers associated with a pathological phenotype [20]–[22], we focused on SMCs-like cells obtained under hypoxia conditions and we checked the preservation of the contractile phenotype after further cell expansion (effect of passage number) and culture even under normoxic conditions.

These experiments demonstrate clearly the deterministic role of the oxygen content in vascular progenitor cells differentiation into mature functional cells constituting the vascular wall (media and intima).

## Methods

### 1) Polyelectrolyte Multilayer Films (PEMs)

PEMs were built with cationic poly (allylamine hydrochloride) (PAH, MW = 70 kDa), and anionic poly(sodium-4-styrene sulfonate) (PSS, MW = 70 kDa) solutions (Sigma-Aldrich, France) as previously described [19], [23]. Briefly, PEMs were prepared on glass coverslips (CML, Nemours, France) pretreated with 0.01 M SDS and 0.12 M HCl for 15 min at 100°C and then extensively rinsed with deionized water. Glass coverslips were deposited in 24-well plates (Nunc, France). PAH-(PSS-PAH)_3_ films were obtained by alternated immersion of the pretreated coverslips for 10 min in polyelectrolyte solutions (300 µL) at 5 mg/mL in the presence of 10 mM Tris-(hydroxymethyl) aminoethane (Tris) and 150 mM NaCl at pH 7.4. After each deposition, the coverslips were rinsed three times during 10 min with 10 mM Tris and 150 mM NaCl at pH 7.4. All the films were sterilized for 10 min by UV light (254 nm).

### 2) Isolation and culture of Mononuclear Cells from peripheral blood circulation

The experimental procedures were used in accordance with the “Principle of Laboratory Animal Care and the Guide for the Care and Use of Laboratory Animals” (National Institute of Health publication No. 80–23, revised 1978). Blood (50 mL) was collected from white New Zealand rabbits (male, average weight 3–3.5 kg, CEGAV, France) carotid into heparinised plastic syringes. Peripheral Blood Mononuclear Cells (MNCs) were isolated using a density gradient as previously described [19]. The cells were then cultivated in endothelial basal medium (EBM-2: Lonza, Belgium) supplemented with angiogenic growth factors (EGM-2-singleQuots® Lonza, Belgium). Cells were counted using Trypan Blue® and were seeded at a density of 1×10^6^ cells/cm^2^ in 24-well plates containing glass coverslips coated either by Type I collagen 1% (BD Biosciences, France) or a PEMs films, made of PSS and PAH (Sigma, France) with a final PAH-(PSS-PAH)_3_ architecture corresponding to 3.5 pairs of deposited PAH/PSS layers [19]. The cultures were placed in normal cell culture incubator at 37°C in an atmosphere with 5% CO_2_ and 21% O_2_, (O_2_/CO_2_ incubator, Sanyo, France). After three days, the medium was removed in order to discard unattached cells. The cells (CD34^+^, CD133^+^ were identified previously [19]) were then placed under hypoxia at 37°C, 5% CO_2_ and 5% O_2_ or under normoxia at 37°C, 5% CO_2_ and 21% O_2_ (control) and medium changed every two days. The differentiation and morphological evolution of the adherent cells were followed by Phase-contrast microscopy observations (Nikon DIAPHOT 300, Japan).

### 3) Immunostaining for smooth muscle cells (SMCs) and endothelial cells (ECs) specific markers

At confluence and after the third passage, cells were also immunolabelled against SMCs and ECs specific markers. Three antibodies were used to characterize the contractile SMCs phenotype: *i*) Alpha Smooth Muscle Actin (α-SMA), *ii*) Smooth Muscle Myosin Heavy Chain (SM-MHC) and *iii*) Calponin. Two other antibodies were used for the ECs phenotype: *i*) CD31 *ii*) von Willebrand factor (vWF) (all from Dako, France). Prior to the immunolabelling with the intracellular antibodies (α-SMA, SM-MHC, Calponin and vWF), the cells were fixed with paraformaldehyde (PAF) 4% (*w/v* in phosphate buffer saline) for 10 min and permeabilized with Triton X-100 0.5% (*w/v* in distilled water) for 15 min. For CD31 labelling the second step (permeabilization) was not performed. The cells were incubated for 45 min at 37°C with the primary monoclonal antibodies, diluted at 1/50 in RPMI 1640 without phenol red, containing bovine serum albumin (BSA 0.5%, *w/v*). After two washes with RPMI 1640, the secondary antibody labelled with Alexa-Fluor® 488 diluted at 1/100 was incubated for 30 min at 37°C. The cells were observed by fluorescence confocal microscopy (LEICA DMIRE2 HC Fluo TCS 1-B, Germany) using the 488 nm spectral line.

### 4) Immunostaining for extracellular matrix (ECM) proteins

At confluence, hypoxia differentiated cells were immunostained for ECM proteins characterization via two specific proteins such as *i*) laminin and *ii*) type IV collagen. The differentiated cells were fixed with PAF 4% for 10 min and incubated for 45 min at 37°C with the primary monoclonal antibodies, diluted at 1/50 in RPMI 1640 without phenol red, containing 0.5% BSA. After two washes with RPMI 1640, the secondary antibody labelled with Alexa-Fluor® 488 diluted at 1/100 was incubated for 30 min at 37°C. The cells were observed using fluorescence confocal microscopy (LEICA DMIRE2 HC Fluo TCS 1-B, Germany).

### 5) Evaluation of the maintenance of the SMCs phenotype

In order to check that after a first step of culture under hypoxia, the differentiation into SMCs was stable versus time, cells were further cultivated either under hypoxia or normoxia. After differentiation the confluent cells cultivated on type I collagen and PEMs were amplified and separated in two batches. The first batch was kept under hypoxic condition (37°C, 5% CO_2_ and 5% O_2_) whereas the second batch was placed in normoxic conditions (37°C, 5% CO_2_ and 21% O_2_). Cells were then cultivated in these different conditions until the third passage (P3) and mature SMCs from rabbit aorta cultivated under the same conditions were used as control.

### 6) Fluorescence Activated Cell Sorting (FACS)

FACS analyses (EPICS XL, Beckman Coulter, France) were performed to quantify the percentage of positive cells and the fluorescence intensity of the specific contractile markers expressed by the differentiated SMCs. After P3, FACS was performed to identify intracellular antigens in cells. For that, trypsinized differentiated cells were labelled as previously described. The non-specific binding was evaluated by the incubation of cells only with the second antibody. Within the differentiated cell area, as determined by forward and sideward scattering, 10,000 events were collected and the percentage of positive cells and the mean fluorescence intensity (MFI) were determined.

### 7) Statistics

The data were expressed as mean±standard error of the mean (s.e.m.) for each condition. Each experiment was repeated in triplicate independently three times. Mean values were compared with the unpaired *t*-test (Statview IV, Abacus Concepts Inc, Berkley, CA, USA), in which *p* represents the rejection level of the null-hypothesis of equal means.

## Results and Discussion

**Figure 1:**
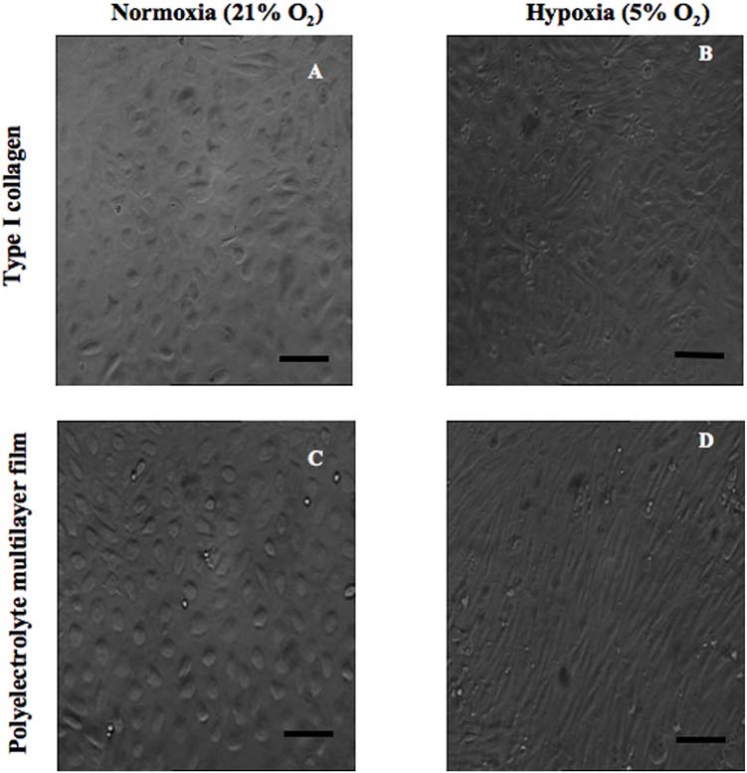
Morphological aspect of differentiated cell. Optical phase contrast microscopy visualization of differentiated cells seeded on type I collagen (A, B) and polyelectrolyte multilayer films (PEMs) (C, D) until confluence under normoxic (A, C) and hypoxic (B, D) environment. Objective×20, scale bar 55 µm. The morphological examination of the confluent cells showed cobblestone shape (A, C) in normoxia and a spindle like (B, D) shape in hypoxia.

Peripheral blood mononuclear cells (MNCs) fraction containing progenitor cells was isolated and seeded in 24-well plates containing glass coverslips coated with type I collagen or with a Polyelectrolyte Multilayer Film (PEMs) at 1×10^6^ cells/cm^2^. We used type I collagen known as an ideal substrate for vascular progenitor cells culture [2] and PEMs for their high potentialities to boost progenitor cell differentiation [19]. After 4 days of culture in normoxic conditions, unattached cells were removed and the adherent cells (CD34^+^, CD133^+^) were divided in two fractions and placed under hypoxia (5% CO_2_ and 5% O_2_) or normoxia (5% CO_2_ and 21% O_2_) until confluence (between 2 and 4 weeks). At confluence and for both surface types, the phase-contrast microscopy cell observation showed cobblestone morphology in normoxic conditions (Figure 1A, 1C) and a spindle like morphology in hypoxic conditions (Figure 1B, 1D).

**Figure 2:**
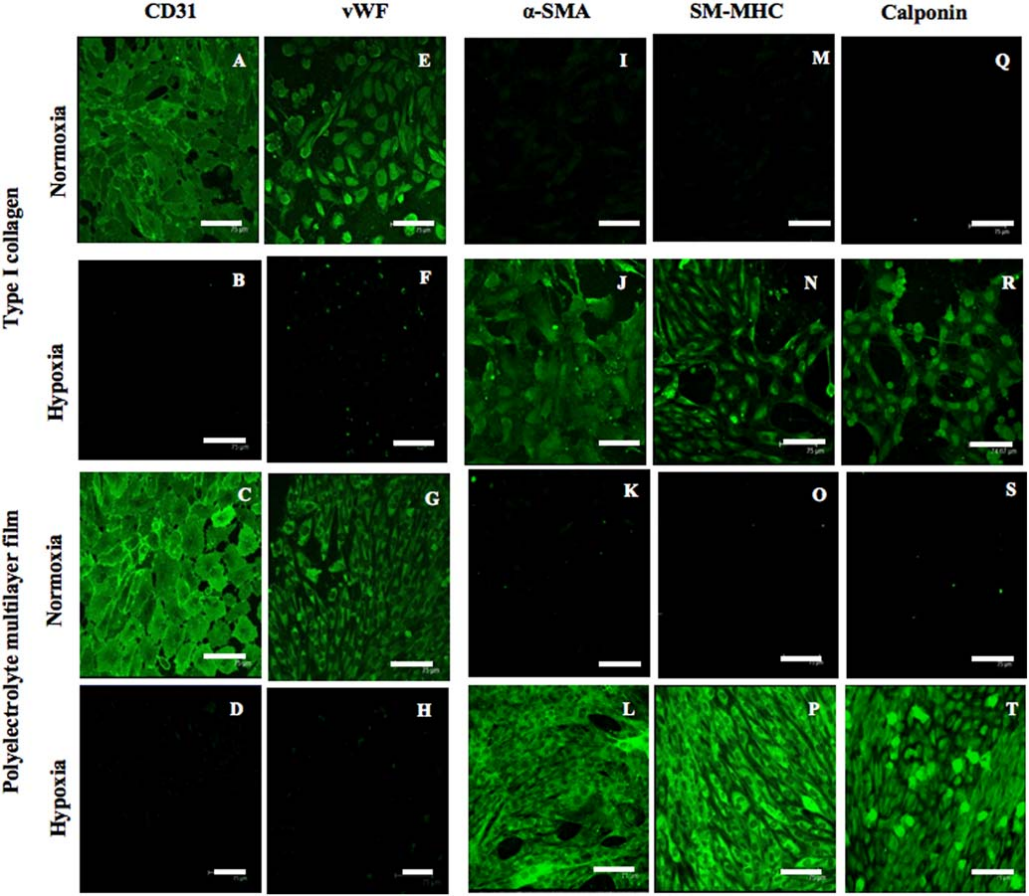
Vascular cell phenotype characterization. The endothelial cell were characterized by the expression of specific markers: CD31 (A–D) and von Willebrand Factor (E–H) and the smooth muscle cells by the expression of contractile markers: α- Smooth Muscle Actin (α-SMA: E–H), Smooth Muscle Myosin Heavy Chain (SM-MHC: I–L) and Calponin (M–P). Images were obtained by confocal microscopy observation at cell confluence on both coated surfaces (type I collagen and Polyelectrolyte Multilayer films (PEMs)) and cultivated under normoxic and hypoxic conditions. Objective×40, NA = 0.8, scale bars 75 µm. The figure showed the positive expression of specific ECs markers for cell differentiated under normoxic environment and positive expression of specific contractile SMCs markers for cell differentiated under hypoxic environment.

In order to evaluate the cell phenotype of differentiated cells, we checked the expression of specific markers of vascular cells (SMCs and ECs) *i.e*. alpha-Smooth Muscle Actin (α-SMA), Smooth Muscle Myosin Heavy Chain (SM-MHC) and Calponin known to assess vascular SMCs differentiation and their contractile function [2], [24], [25] and CD31 and von Willebrand Factor (vWF) for the ECs phenotype evaluation [26], [27]. As expected under normoxic conditions, the confocal microscopy observations showed the presence of positive cells for ECs markers [Figure 2A and 2E (for type I collagen coating), 2C and 2G (for PEMs coating)] and negative cells for SMCs markers [Figure 2I, 2M and 2Q (for type I collagen), 2K, 2O and 2S (for PEMs)]. Under hypoxia a surprising positive expression of SMCs markers was observed [Figure 2J, 2N and 2R (for type I collagen), 2L, 2P and 2T (for PEMs)]. No expression of ECs markers was noticed under this condition whatever the surface coating [Figure 2B and 2F (for Type I collagen), Figure 2D and 2H (for PEMs)] indicating thus a total absence of cellular differentiation into ECs at a low concentration of O_2_. All these observations constitute a signature for the progenitor cells switching into SMCs phenotype. These results suggest first the potentiality of MNCs cells to differentiate into a SMCs phenotype under a hypoxic environment and second the expression of the specific markers confirmed the contractile phenotype of these cells [28] (similar to SMCs *in vivo*). In the literature the hematopoietic stem cells differentiation into mature and functional SMCs requires the culture medium supplementation with specific growth factors, especially PDGF-BB [2], [3]. Our results demonstrate that the oxygen concentration tuning alone allows phenotype switch either to endothelial cells or smooth muscle cells.

**Figure 3:**
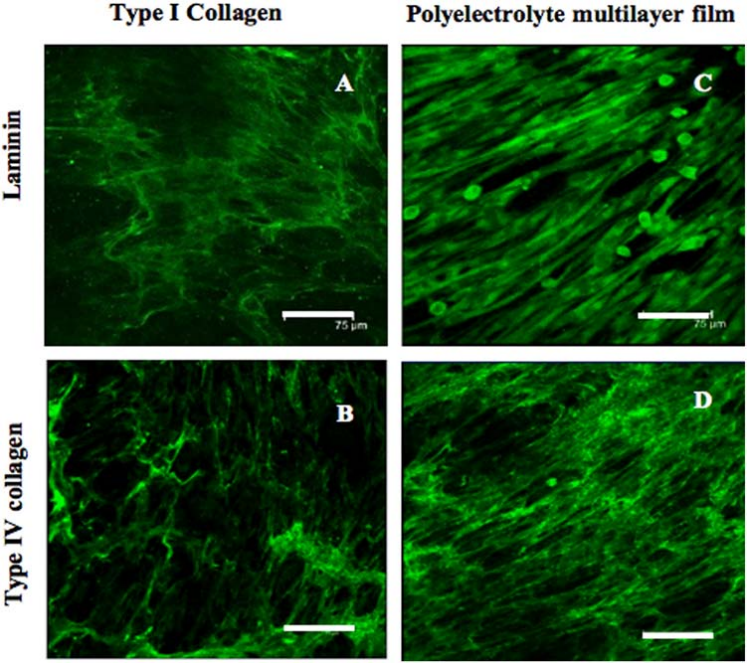
Extracellular matrix synthesis. The confocal microscopy observations of Extracellular matrix protein: laminin (A, B) and type IV collagen (C, D) of cells seeded on both coated surfaces (type I collagen and Polyelectrolyte Multilayer films (PEMs)). Objective×40, NA = 0.8, scale bars 75 µm.

The extracellular matrix (ECM) contributes to the control of the cellular function and is involved in maintaining the cells in a differentiated state [29], [30]. During blood vessel formation the SMCs are responsible for extracellular matrix formation *via* protein (fibronectin, laminin, collagens…) secretion [31]. The ECM deposition contributes in vivo and in vitro (tissue engineering approach) to arterial wall constitution and cell function via different signalling pathways (kinase pathways activation) [31], [32]. We investigated the capacity of the differentiated cells under hypoxic conditions to synthesize their own ECM, and we evaluated the secretion of two extracellular proteins (Laminin and type IV collagen), which play a major role in ECM synthesis and contribute to maintain the contractile phenotype of the differentiated cells [31]. Confocal microscopy observations showed the deposition of both of these proteins. The comparison between both surfaces showed moreover a stronger synthesis of ECM by the cells cultivated on PEMs (Figure 3). These data obtained under hypoxic conditions confirmed the capacity of MNCs to differentiate into SMCs, exhibiting a contractile phenotype, sign of a correct physiological state and integrity of the ECM. This integrity plays a key role to maintain this state and suggests stability over longer time periods.

The phenotype stability over a longer time period of the SMCs derived from MNCs cultivated under hypoxia is a major issue to use this route in tissue engineering for example. The SMCs phenotype stability was investigated at low or high oxygen concentration. After the first passage of hypoxic differentiated cells (cells positive to SMCs markers), the obtained cells were expanded under two conditions. For the first assay we maintained cells under hypoxic condition and for the second assay we placed cells in normoxic condition. In order to check the stability of the SMCs phenotype under these conditions, several passages (P3) were performed. Whatever the experimental condition (hypoxic and normoxic conditions) we never detected ECs markers (data not shown).

**Figure 4:**
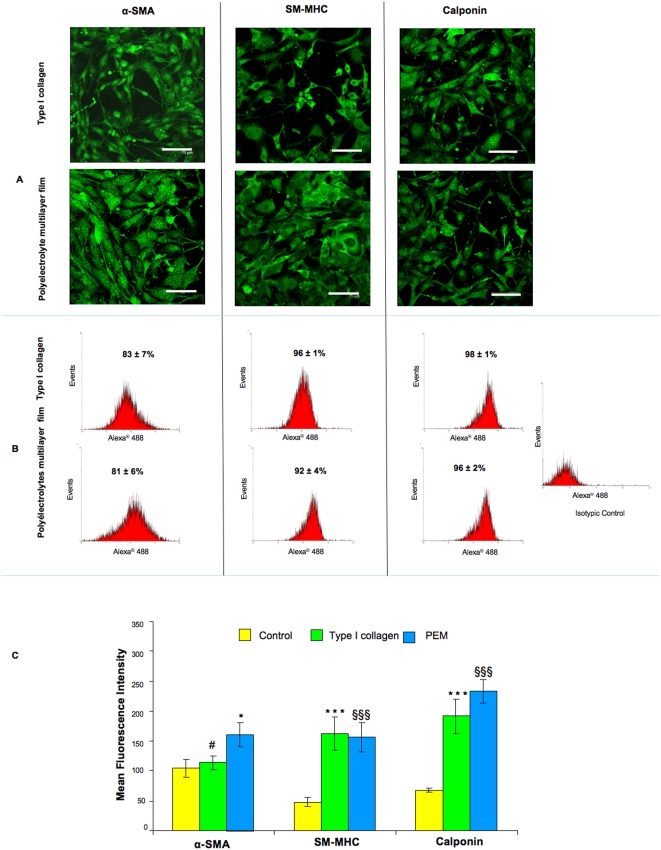
Phenotype stability under hypoxia. After the third passage, the smooth muscle cells phenotype stability of differentiated cell cultivated under hypoxic conditions was investigated by confocal microscopy observation (A) and flow cytometry analyses (B, C). A: Confocal microscopic observations showed positive cells for contractile markers: α- Smooth Muscle Actin (α-SMA), Smooth Muscle Myosin Heavy Chain (SM-MHC) and Calponin confluence on both coated surfaces (type I collagen and Polyelectrolyte Multilayer films (PEMs)). Objective×40, NA = 0.8, scale bars 75 µm. B: Flow cytometry showed that more than 80% cells expressed SMCs markers. C: Mean fluorescence intensity analyses showed a higher SMCs contractile markers expression for differentiated cells compared to control (mature SMCs) whatever the surface coating. (§)PEMs *versus* control, (*) Collagen *versus* control, (#) PEMs *versus* collagen. (§,* and #: *p*<0.05 and §§§ and ***: *p*<0.001).

Under hypoxia the cell characterization showed the positive staining for SMCs markers with a regular cytosolic distribution of all observed SMCs markers (Figure 4A) for both coating types (Type I collagen and PEMs). These data were correlated with FACS analyses which indicated that, after the third passage, more than 80% of cells were positive for both surfaces (Figure 4B). We compared moreover the Mean Fluorescence Intensity (MFI) of SMCs contractile markers expression of the differentiated cells with mature SMCs extracted from rabbit aorta and cultivated in the same medium in normoxic and hypoxic conditions. Mature SMCs were cultivated on the usually employed tissue culture plastic surface (TCPS) [33] showing no difference with a control performed on type I collagen and PEMs. The expression of α-SMA, SM-MHC and calponin for cells cultivated on both Type I collagen and PEM coated surfaces was significatively higher for the differentiated cells compared to mature SMCs, although less important on the collagen coated surface for α-SMA.

**Figure 5:**
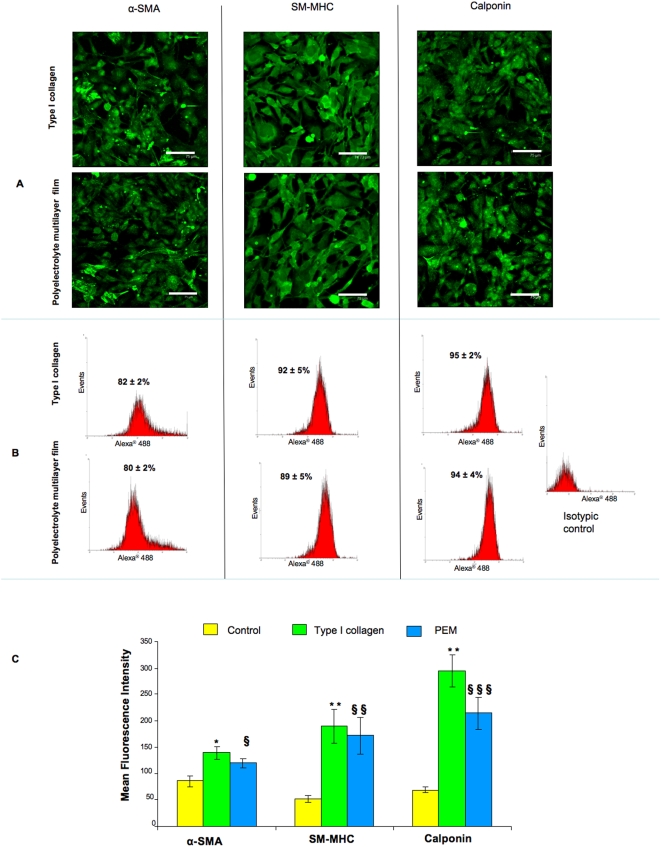
Phenotype stability under normoxia. After the third passage, the smooth muscle cells phenotype stability of differentiated cell cultivated under normoxic conditions was investigated by confocal microscopy observation (A) and flow cytometry analyses (B, C). A: Microscopical observations show positive cells for contractile markers: α- Smooth Muscle Actin (α-SMA), Smooth Muscle Myosin Heavy Chain (SM-MHC) and Calponin confluence on both coated surfaces (type I collagen and Polyelectrolyte Multilayer films (PEMs)). Objective×40, NA = 0.8, scale bars 75 µm. B: Flow cytometry showed that about 90% cells expressed SMCs markers. C: Mean fluorescence intensity analyses showed a higher SMCs contractile markers expression for differentiated cells compared to control (mature SMCs) whatever the surface coating. (§) PEMs *versus* control, (*) Collagen *versus* control. (§ and *: *p*<0.05, §§ and **: *p*<0.01, and *** *p*<0.001).

Under normoxic conditions, the expanded cells were also qualitatively and quantitatively characterized by confocal microscopy observations and by FACS analyses. As for hypoxic conditions, the visualized cells were positive for SMCs contractile markers with again a regular cytoplasmic distribution (Figure 5A). FACS analyses showed also that more than 80% of differentiated cells were positive to SMCs contractile markers (Figure 5B). The MFI of contractile markers for differentiated cells was significatively higher than for mature SMCs for both surfaces coating and with no differences for differentiated cells cultivated on type I collagen and PEMs coated surfaces (Figure 5C). It is also important to state that no significatively difference was found in the expression of the three contractile markers once comparing the data obtained in hypoxic and normoxic conditions.

It is well known that *in vitro* mature SMCs extracted from vessels switch their phenotype from a contractile (healthy) to a proliferative (pathological) phenotype [34], [35]. This switch constitutes a strong limitation for blood vessel tissue engineering. The present differentiation approach allowed us to obtain a “healthy” phenotype of SMCs which could constitute an alternative for vascular tissue engineering. We observed effectively a quite stronger expression of the contractile markers for the differentiated cells compared to mature SMCs. In a different context (in particular during vascular wall remodelling after bypass surgery) a reduced tissue oxygenation together with the presence of inflammatory cells could be at the origin of vascular wall recolonization by SMCs, the origin of which was not elucidated up to now [9], [36]–[38].

To conclude we demonstrated that progenitor cells cultivated in hypoxic conditions and without specific growth factor enhancing SMCs differentiation displayed morphological and phenotypic properties of SMCs as showed by the expression of SMCs contractile markers. Moreover, these differentiated SMCs maintained their contractile phenotype when replaced in normoxic conditions suggesting that these cells developed a stable and functional phenotype comparable to physiological SMCs found in functional blood vessels.

These results highlight the crucial role of the tissue environment and especially the O_2_ content in the differentiation process of vascular progenitor cells. These observations combined with previous ones [19] could constitute a basis for tissue engineering and clinical application strategies for *in vitro* tissue reconstruction. For example in vascular tissue engineering, starting from an unique peripherical blood sample cultivated on PEM and with the same culture media, but in normoxic or in hypoxic conditions either mature ECs (21% O_2_) or contractile SMCs (5% O_2_) can be obtained in less than one month. The different layers (media and intima) could be associated to build for example a natural a natural and autologous vascular graft.

## References

[1] Asahara T, Murohara T, Sullivan A, Silver M, van der Zee R (1997). Isolation of putative progenitor endothelial cells for angiogenesis.. Science.

[2] Simper D, Stalboerger PG, Panetta CJ, Wang S, Caplice NM (2002). Smooth muscle progenitor cells in human blood.. Circulation.

[3] Xie SZ, Fang NT, Liu S, Zhou P, Zhang Y (2008). Differentiation of smooth muscle progenitor cells in peripheral blood and its application in tissue engineered blood vessels.. J Zhejiang Univ Sci B.

[4] Liu JY, Swartz DD, Peng HF, Gugino SF, Russell JA (2007). Functional tissue-engineered blood vessels from bone marrow progenitor cells.. Cardiovasc Res.

[5] Yeh ET, Zhang S, Wu HD, Körbling M, Willerson JT (2003). Transdifferentiation of human peripheral blood CD34+-enriched cell population into cardiomyocytes, endothelial cells, and smooth muscle cells in vivo.. Circulation.

[6] Takahashi T, Kalka C, Masuda H, Chen D, Silver M (1999). Ischemia- and cytokine-induced mobilization of bone marrow-derived endothelial progenitor cells for neovascularization.. Nat Med.

[7] Davie NJ, Crossno JT, Frid MG, Hofmeister SE, Reeves JT (2004). Hypoxia-induced pulmonary artery adventitial remodeling and neovascularization: contribution of progenitor cells.. Am J Physiol Lung Cell Mol Physiol.

[8] Stenmark KR, Fagan KA, Frid MG (2006). Hypoxia-induced pulmonary vascular remodeling: cellular and molecular mechanisms.. Circ Res.

[9] Kerdjoudj H, Berthelemy N, Rinckenbach S, Kearney-Schwartz A, Montagne K (2008). Small vessel replacement by human umbilical arteries with polyelectrolyte film-treated arteries: in vivo behavior.. J Am Coll Cardiol.

[10] Falanga V, Qian SW, Danielpour D, Katz MH, Roberts AB (1991). Hypoxia upregulates the synthesis of TGF-beta 1 by human dermal fibroblasts.. J Invest Dermatol.

[11] Payne TR, Oshima H, Okada M, Momoi N, Tobita K (2007). A relationship between vascular endothelial growth factor, angiogenesis, and cardiac repair after muscle stem cell transplantation into ischemic hearts.. J Am Coll Cardiol.

[12] Cramer T, Schipani E, Johnson RS, Swoboda B, Pfander D (2004). Expression of VEGF isoforms by epiphyseal chondrocytes during low-oxygen tension is HIF-1 alpha dependent.. Osteoarthritis Cartilage.

[13] Conway EM, Collen D, Carmeliet P (2001). Molecular mechanisms of blood vessel growth.. Cardiovasc Res.

[14] Grayson WL, Zhao F, Izadpanah R, Bunnell B, Ma T (2006). Effects of hypoxia on human mesenchymal stem cell expansion and plasticity in 3D constructs.. J Cell Physiol.

[15] Malda J, Klein TJ, Upton Z (2007). The roles of hypoxia in the in vitro engineering of tissues.. Tissue Eng.

[16] Simon MC, Keith B (2008). The role of oxygen availability in embryonic development and stem cell function.. Nat Rev Mol Cell Biol.

[17] Gerasimovskaya EV, Woodward HN, Tucker DA, Stenmark KR (2008). Extracellular ATP is a pro-angiogenic factor for pulmonary artery vasa vasorum endothelial cells.. Angiogenesis.

[18] Griese DP, Ehsan A, Melo LG, Kong D, Zhang L (2003). Isolation and transplantation of autologous circulating endothelial cells into denuded vessels and prosthetic grafts: implications for cell-based vascular therapy.. Circulation.

[19] Berthelemy N, Kerdjoudj H, Gaucher C, Schaaf P, Stoltz JF (2008). Polyelectrolyte Films boost Progenitor Cell Differentiation into Endothelium-like Monolayers.. Adv Mater.

[20] Reusch P, Wagdy H, Reusch R, Wilson E, Ives HE (1996). Mechanical strain increases smooth muscle and decreases non-muscle myosin expression in rat vascular smooth muscle cells.. Circ Res.

[21] Rovner AS, Murphy RA, Owens GK (1986). Expression of smooth muscle and non-smooth muscle myosin heavy chains in cultured vascular smooth muscle cells.. J Biol Chem.

[22] Muto A, Fitzgerald TN, Pimiento JM, Maloney SP, Teso D (2007). Smooth muscle cell signal transduction: implications of vascular biology for vascular surgeons.. J Vasc Surg.

[23] Kerdjoudj H, Boura C, Moby V, Montagne K, Schaaf P (2007). Re-endothelialization of Human Umbilical Arteries Treated with Polyelectrolyte Multilayers: A Tool for Damaged Vessel Replacement.. Adv Func Mater.

[24] Babu GJ, Pyne GJ, Zhou Y, Okwuchukuasanya C, Brayden JE (2004). Isoform switching from SM-B to SM-A myosin results in decreased contractility and altered expression of thin filament regulatory proteins.. Am J Physiol Cell Physiol.

[25] Li S, Fan YS, Chow LH, Van Den Diepstraten C, van Der Veer E (2001). Innate diversity of adult human arterial smooth muscle cells: cloning of distinct subtypes from the internal thoracic artery.. Circ Res.

[26] Newman PJ, Berndt MC, Gorski J, White GC, Lyman S (1990). PECAM-1 (CD31) cloning and relation to adhesion molecules of the immunoglobulin gene superfamily.. Science.

[27] Meyer D, Piétu G, Fressinaud E, Girma JP (1991). von Willebrand factor: structure and function.. Mayo Clin Proc.

[28] Owens GK (1995). Regulation of differentiation of vascular smooth muscle cells.. Physiol Rev.

[29] Ingber DE, Dike L, Hansen L, Karp S, Liley H (1994). Cellular tensegrity: exploring how mechanical changes in the cytoskeleton regulate cell growth, migration, and tissue pattern during morphogenesis.. Int Rev Cytol.

[30] Bissell MJ, Barcellos-Hoff MH (1987). The influence of extracellular matrix on gene expression: is structure the message?. J Cell Sci.

[31] Rzucidlo EM, Martin KA, Powell RJ (2007). Regulation of vascular smooth muscle cell differentiation.. J Vasc Surg.

[32] Davis MJ, Wu X, Nurkiewicz TR, Kawasaki J, Davis GE (2001). Integrins and mechanotransduction of the vascular myogenic response.. Am J Physiol Heart Circ Physiol.

[33] L'Heureux N, Stoclet JC, Auger FA, Lagaud GJ, Germain L (2001). A human tissue-engineered vascular media: a new model for pharmacological studies of contractile responses.. FASEB J.

[34] Cha JM, Park SN, Noh SH, Suh H (2005). Time-dependent modulation of alignment and differentiation of smooth muscle cells seeded on a porous substrate undergoing cyclic mechanical strain.. Artif Organs.

[35] Bach AD, Stem-Straeter J, Beier JP, Bannasch H, Stark GB (2003). Engineering of muscle tissue.. Clin Plast Surg.

[36] Chaouat M, Le Visage C, Autissier A, Chaubet F, Letourneur D (2006). The evaluation of a small-diameter polysaccharide-based arterial graft in rats.. Biomaterials.

[37] Mellander S, Fogelstrand P, Enocson K, Johansson BR, Mattsson E (2005). Healing of PTFE grafts in a pig model recruit neointimal cells from different sources and do not endothelialize.. Eur J Vasc Endovasc Surg.

[38] L'Heureux N, Dusserre N, Konig G, Victor B, Keire P (2006). Human tissue-engineered blood vessels for adult arterial revascularization.. Nat Med.

